# Plexiform Schwannoma of the Penis: A Rare Subtype of Genital Schwannoma

**DOI:** 10.1155/2019/1752314

**Published:** 2019-04-03

**Authors:** Chrysovalantis Gkekas, Vasileios Kalyvas, Evangelos N. Symeonidis, Apostolos Malioris, Michail Papathanasiou, Nikolaos Kalinderis, Kyriakos Moisidis, Konstantinos Hatzimouratidis

**Affiliations:** ^1^Department of Urology, 424 General Military Hospital of Thessaloniki, Thessaloniki, Greece; ^2^2nd Department of Urology, Aristotle University of Thessaloniki, Thessaloniki, Greece

## Abstract

Schwannomas are benign, encapsulated neurogenic tumors which present in diverse histological subtypes despite the limited variety of their cellular constituents. These include the cellular, ancient, cystic, epithelioid, melanotic, psammomatous, schwannoma with pseudoglandular elements, and plexiform varieties. The plexiform schwannoma (PS) represents 4.3% of all schwannomas. These lesions are commonly encountered in the head and neck region and are extremely rare in the penis. To the best of our knowledge only 34 cases of penile schwannomas have been reported and this is the 3rd case of plexiform penile schwannoma. A 39-year-old patient presented to our andrology outpatient clinic complaining for two painful penile nodules. The lesions were located on the dorsum of the penile shaft. His medical history was insignificant for penile trauma and sexual transmitted diseases. The masses measured 2x1 cm and 0.5x1 cm. After sonographic and magnetic resonance evaluation the patient was admitted to theatre and underwent topical resection of the lesions. Histopathology revealed plexiform schwannoma. Postoperatively, penile tenderness and hyperesthesia ensued which was managed with pregabalin administration and topical corticosteroids. Plexiform schwannomas are rare in the penile region. Surgical excision is inevitably the only way to diagnose and treat the lesions. They must be differentiated by a variety of malignant and benign clinical conditions. Topical excision suffices for oncological control and allows for acceptable functional outcomes.

## 1. Background

Plexiform schwannomas (PS) represent a rare subtype of schwannomas. External genitalia are a relatively unusual landing location for this type of lesions and can pose a diagnostic and therapeutic challenge. We hereby report the case of a 39-year-old patient with penile PS, its diagnostic approach, and definitive treatment.

## 2. Introduction

Schwannomas are benign, encapsulated neurogenic tumors originating from the Schwann cells of the peripheral nerve sheath [1]. Plexiform schwannoma represents a unique variant, accounting for 4.3% of all schwannomas. These lesions are commonly encountered in the head and neck region [2]. Despite the extensive innervation of genitalia, neurilemmomas (Schwannomas) are exceedingly rare in the penis. Two recent reviews have identified 34 cases of penile schwannomas published in the English literature and this represents the 3rd characterized PS variant isolated to the penis [3–5].

Although their benign behavior is a fundamental feature of PS they can easily be confused with plexiform neurofibromas. The latter are pathognomonic of Von Recklinghausen's disease and have malignant potential [4, 6].

Plexiform schwannomas on the contrary can occur even in the absence of Von Reckligausen's genotype and malignant transformation has never been described [6].

## 3. Case Presentation

**Figure 1:**
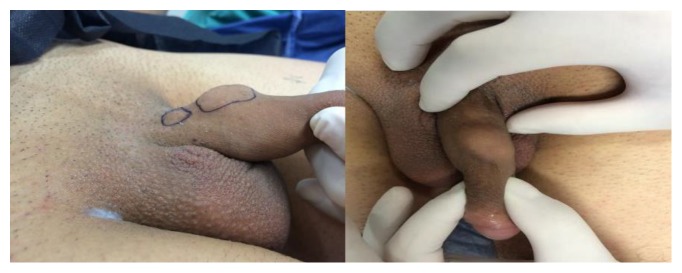
Two separate lumps on the dorsolateral aspect of the penis.

**Figure 2:**
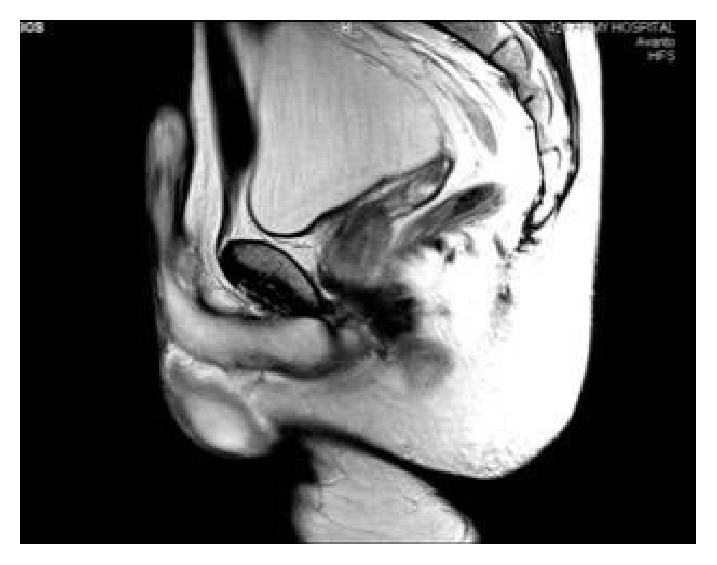
MRI of the penis T2 sagittal, demonstrating a distinct nodule on the dorsal aspect of the penis.

**Figure 3:**
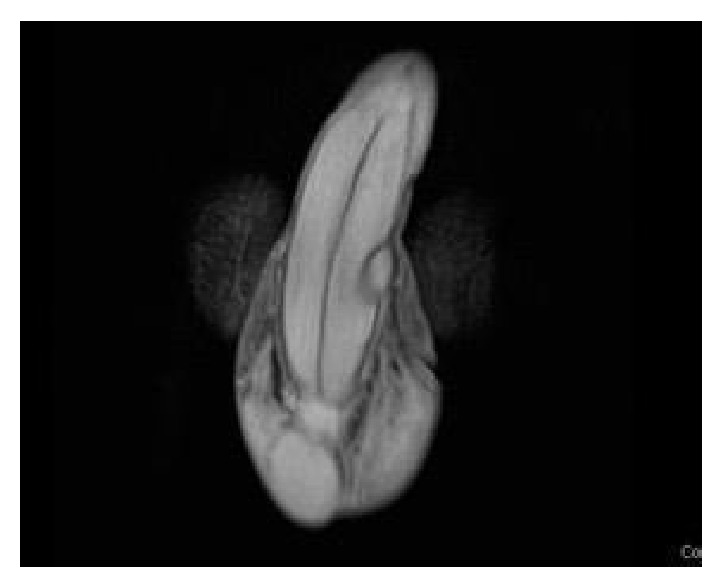
MRI of the penis T2 coronal. Dorsolaterally located nodule: clearly separate from tunica albuginea but included in Buck's fascia.

A 39-year-old patient presented to our andrology clinic complaining of two gradually enlarging penile lumps. The lesions were located on the dorsum of the penile shaft having enlarged within a 2-year period. He complained of mild dorsal deviation of the shaft on erection and reported occasional pain during intercourse. He had not had any history of penile trauma or any sexually transmitted disease. Clinically, two distinct, soft, and painless nodules with a smooth contour could be identified on the dorsum of the penis. They were not fixed in place, yet not completely mobile (Figure 1). Artificial erection excluded penile curvature. Blood tests and urinalysis were insignificant. Sonographically, the lesions appeared hypoechoic and contained within a hyperechoic capsule, exhibiting diffuse central and peripheral blood flow. Magnetic resonance imaging depicted two well-capsulated masses ensheathed within Buck's fascia measuring 2x1 cm and 0.5x1cm each (Figures 2 and 3).

**Figure 4:**
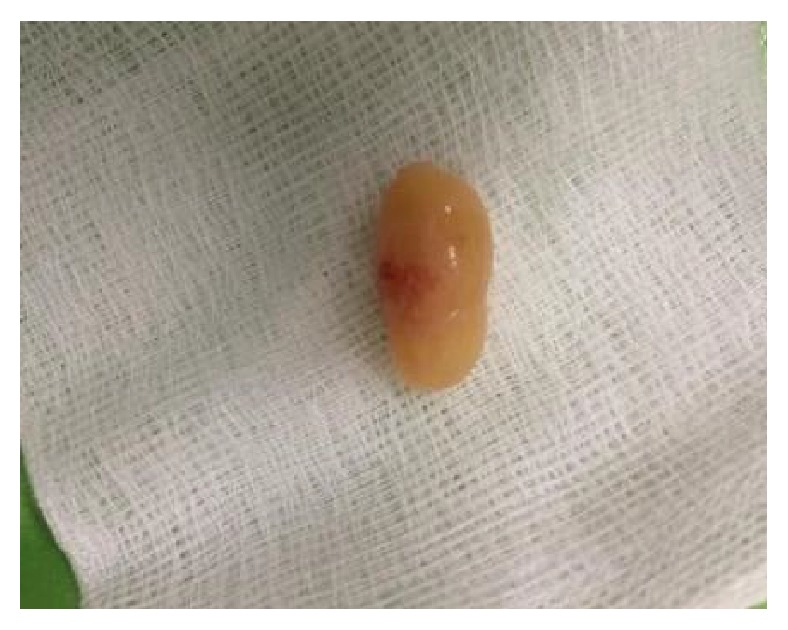
Penile plexiform schwannoma. Surgical specimen after excision: yellowish, spindle-shaped, and elastic in consistency.

**Figure 5:**
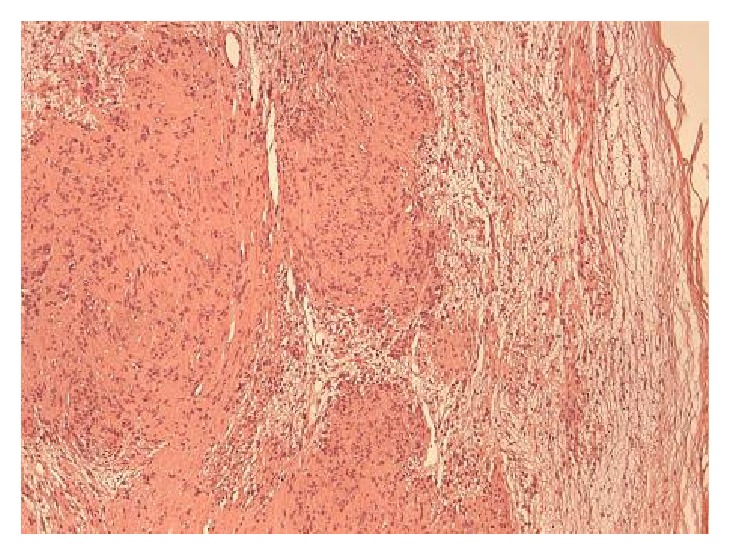
Small plexiform nodules with elongated, spindle-shaped tumor cells with no atypia or mitoses: Antoni A (left). Haphazard, scarce cellular arrangement in a loose stroma: Antoni B (right). E&H stain x10.

**Figure 6:**
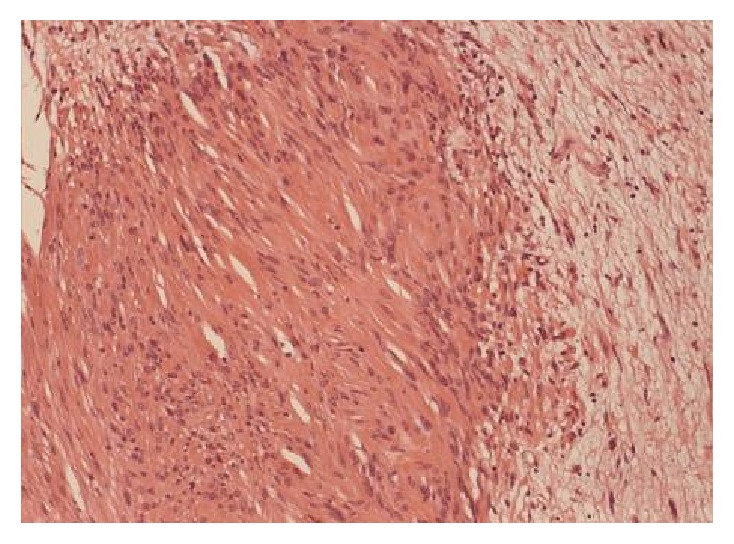
Antoni A area on the left with dense cellularity and nuclear palisading. Antoni B area on the right demonstrating loose array of the cells. E&H stain x20.

The larger and peripherally located lesion was removed via a 2 cm dorsal midline shaft incision under local anesthetic infiltration and penile block. The incision was restricted to the dorsal shaft without the need for circumcision. Macroscopically, the specimen appeared to be a 2.3cm spindle-shaped, elastic mass (Figure 4). In cross-section it had a soft, elastic texture and a yellow-tanned tinge. Histopathology reported a PS with strongly positive S-100 immunostain consistent with the neural origin of the tumor cells, the characteristic Antoni A and B areas, and the nuclear palisading (Figures 5 and 6). At this point it is worth mentioning that the patient had no physical findings of neurofibromatosis (NF), i.e., no café-au-lait spots, no Lisch nodules, no freckles, no osseous malformations, and no family history of NF.

Postoperatively, there was tenderness of the dorsal shaft and granular hyperesthesia with intermittent tingling and burning sensation. The symptoms attenuated after 6 months upon administration of low dose oral pregabalin and topical corticosteroids. This combination treatment was deemed necessary after the initial failure of simple analgesics for the relief of the neuropathic penile pain resulting from surgery and postop scarring. The patient returned to his preoperative sexual status at 6 months after the operation and upon resolution of his numbness and dysesthesia.

Nine months after the removal of the first lesion the patient underwent excision of the second and more proximal lesion. It was less mobile and firmly attached to the neurovascular bundle and due to its proximal position was marginally out of the area covered by the penile block. It was removed under general anesthesia. Histology confirmed multifocal PS and due to its firm attachment to the neurovascular bundle the patient experienced dysesthesia of the glans and developed a dorsal cicatricial band causing discomfort during erection. Once again it was treated with pregabalin and long-term topical steroids with slow improvement.

## 4. Discussion

Harkin et al. reported the first 6 cases of plexiform schwannoma in 1978 and distinguished them from plexiform neurofibroma [7]. Most lesions were solitary and sporadic. Multifocality was usually associated with neurofibromatosis type 2. Despite PS being considered benign, recurrences have been reported in cases of incomplete resection [7]. They are usually located in the head and neck region (23%), the upper (22%) and lower extremities (22%), and the trunk (18%) [2].

Penile plexiform schwannomas are extraordinarily rare with only 2 cases having been identified so far out of the 34 published cases of penile schwannomas. Recently, in 2017, Kumar U et al. reported an uncommon presentation of sizeable penile schwannoma mimicking a scrotal mass in a 16-year-old boy [8].

Despite considered painless, penile schwannomas have been associated with painful intercourse, erectile dysfunction, and penile curvature[2]. Differential diagnosis includes Peyronie's disease, fibrosis after intracavernosal injections, lipomas, leiomyomas, sarcomas, and the other schwannoma subtypes [9]. Histopathology is the only safe means to distinguish them from other benign lesions [10, 11]. They demonstrate the key histopathological features of schwannomas such as the densely cellular Antoni A areas where nuclear palisading and whorling patterns predominate and the hypocellular Antoni B areas with the loose hypocellular stroma. Plexiform schwannomas are usually superficial, occurring typically in the dermis and subcutaneous tissue.

Malignant peripheral nerve sheath tumors (MPNST) have been described in the penis and require wide excision due to their risk of recurrence and their aggressive behavior [11, 12]. Although plexiform schwannomas have not been associated with malignant transformation, plexiform neurofibromas can progress into MPSNT and therefore identifying PS is essential due to their different biological behavior. S-100 immunostaining could be helpful since it is weakly expressed (or even not at all) in MPNSTs whilst PSs exhibit strong expression of the specific protein. Another differentiating feature is the increased number of mitoses and cellular atypia observed in MPNSTs in comparison to PSs as well as the fact that they are usually deep seated rather than superficial which is the case for most PSs.

Sexual dysfunction is the patient's primary concern and should be taken into consideration in the decision making. However, extensive surgery such as penectomy may be required in patients with advanced malignant disease along with adjuvant radiotherapy [3, 11, 12]. Surgical excision is the only method to differentiate and treat the condition. The rarity of the specific entity and the lack of extensive experience impede the construction of an evidence based treatment algorithm. Every reported case adds on the growing body of evidence.
